# Sequence-based typing of genetic targets encoded outside of the O-antigen gene cluster is indicative of Shiga toxin-producing *Escherichia coli* serogroup lineages

**DOI:** 10.1099/jmm.0.47053-0

**Published:** 2007-05

**Authors:** Matthew W. Gilmour, Adam B. Olson, Ashleigh K. Andrysiak, Lai-King Ng, Linda Chui

**Affiliations:** 1 National Microbiology Laboratory, Public Health Agency of Canada, 1015 Arlington Street, Winnipeg, Manitoba R3E 3R2, Canada; 2 Department of Medical Microbiology and Infectious Diseases, University of Manitoba, Winnipeg, Manitoba, Canada; 3 Alberta Provincial Laboratory for Public Health, Edmonton, Alberta, Canada

## Abstract

Serogroup classifications based upon the O-somatic antigen of Shiga toxin-producing *Escherichia coli* (STEC) provide significant epidemiological information on clinical isolates. Each O-antigen determinant is encoded by a unique cluster of genes present between the *gnd* and *galF* chromosomal genes. Alternatively, serogroup-specific polymorphisms might be encoded in loci that are encoded outside of the O-antigen gene cluster. Segments of the core bacterial loci *mdh*, *gnd*, *gcl*, *ppk*, *metA*, *ftsZ*, *relA* and *metG* for 30 O26 STEC strains have previously been sequenced, and comparative analyses to O157 distinguished these two serogroups. To screen these loci for serogroup-specific traits within a broader range of clinically significant serogroups, DNA sequences were obtained for 19 strains of 10 additional STEC serogroups. Unique alleles were observed at the *gnd* locus for each examined STEC serogroup, and this correlation persisted when comparative analyses were extended to 144 *gnd* sequences from 26 O-serogroups (comprising 42 O : H-serotypes). These included O157, O121, O103, O26, O5 : non-motile (NM), O145 : NM, O113 : H21, O111 : NM and O117 : H7 STEC; and furthermore, non-toxin encoding O157, O26, O55, O6 and O117 strains encoded distinct *gnd* alleles compared to STEC strains of the same serogroup. DNA sequencing of a 643 bp region of *gnd* was, therefore, sufficient to minimally determine the O-antigen of STEC through molecular means, and the location of *gnd* next to the O-antigen gene cluster offered additional support for the co-inheritance of these determinants. The *gnd* DNA sequence-based serogrouping method could improve the typing capabilities for STEC in clinical laboratories, and was used successfully to characterize O121 : H19, O26 : H11 and O177 : NM clinical isolates prior to serological confirmation during outbreak investigations.

## INTRODUCTION

Shiga toxin-producing *Escherichia coli* (STEC) are bacterial pathogens that result in both outbreak and sporadic occurrences of human mortality and disease. Symptoms can include bloody and non-bloody diarrhoea, and children are susceptible to renal failure due to haemolytic uraemic syndrome. STEC are transmitted to humans by consumption of contaminated food or water, person-to-person contact or animal-to-person contact, where natural reservoirs include cattle, pigs and sheep (Karch *et al.*, 2005). Serogroup classifications based upon the O-somatic or H-flagellar antigens of STEC provide significant epidemiological information on clinical isolates, and this measure can provide the first indication of relatedness between strains during outbreak investigations. The serogroup is also indicative of the overall genetic relatedness between *E. coli* strains, including virulence gene content, such as the locus for enterocyte effacement (LEE) pathogenicity island, and the *stx1* and *stx2* loci encoding Shiga toxins (Prager *et al.*, 2005; Girardeau *et al.*, 2005; Karmali *et al.*, 2003).

The predominant O-serogroup of STEC that is observed clinically in North America is O157 (Johnson *et al.*, 2006); however, biased sampling likely results from the availability of clinical media and detection reagents that target this serogroup. Directed studies for the isolation and characterization of both O157 and non-O157 STEC from clinical samples have indicated that the proportion of non-O157 in North America is likely higher than clinical records have indicated (Thompson *et al.*, 2005; Jelacic *et al.*, 2003; Fey *et al.*, 2000). In Canada, over 90 % of STEC strains detected are serotype O157 : H7 or O157 : non-motile (NM) (Woodward *et al.*, 2002). The global prevalence of non-O157 includes significant outbreaks of O26, O121, O103, O111 and O145, and in some countries it is recognized that these serogroups exceed the prevalence of O157 STEC (Karch *et al.*, 2005). Furthermore, non-O157 strains have been identified along with O157 strains in clinical samples (Paton *et al.*, 1996), so it is possible that a diagnostic bias towards O157 may prevent the detection of the aetiological STEC serogroup during human illness.

Molecular methods for the characterization and identification of O-antigen determinants have been devised using restriction profiling and allele-specific PCR. The entire O-antigen-encoding gene cluster could be amplified using primers that targeted conserved regions in the neighbouring *gnd* sequence (encoding 6-phosphogluconate dehydrogenase) and JUMPstart sequence, and enzymic digestion of this amplicon identified RFLPs correlating to O-antigen determinants (Coimbra *et al.*, 2000). This method was problematic due to the length of the amplicon (upwards of 20 kbp) and the absence of unique restriction profiles for all serotypes. Within the O-antigen gene cluster the *wzx* and *wzy* loci encode the O-antigen flippase and polymerase, respectively, and distinct alleles corresponding to each O-serogroup have been used for molecular serogrouping of O103, O157, O26, O113 and O111 strains (Perelle *et al.*, 2005; DebRoy *et al.*, 2004; Paton & Paton, 1999a; Fratamico *et al.*, 2005; D'Souza *et al.*, 2002). It has been suggested that these assays could replace traditional serological methods (DebRoy *et al.*, 2005); however, the individual tests currently detect only one to three O-serogroups. In the absence of a priori knowledge of a serogroup, a large number of reagents may be required to confirm serogroup identity with these methods. Robust platforms such as DNA microarrays containing *wzx* and *wzy* probes targeting up to four *E. coli* serogroups are currently being investigated (Liu & Fratamico, 2006), and broad subtyping of STEC has been achieved using allelic variants of a LEE-encoded determinant (Gilmour *et al.*, 2006).

Multilocus sequence typing has been attempted for each of the STEC serotypes O26 : H11, O121 : H19, O103 : H2 or O157 : H7, but this method was not appropriate for subtyping because very few polymorphisms were observed between strains of the same serotype (Gilmour *et al.*, 2005; Tarr *et al.*, 2002; Noller *et al.*, 2003; Beutin *et al.*, 2005). The genetic differentiation and subtyping of *E. coli* serotype O26 : H11 was attempted by sequencing 10 loci for 30 strains encoding *stx1*, or both *stx1* and *stx2* (Gilmour *et al.*, 2005). Amongst the O26 : H11 strains all loci were identical, with the exception of three alleles of *mdh* and two alleles of *ppk* that each differed by a single point mutation. Notably, comparative analyses of the *mdh*, *gnd*, *gcl*, *ppk*, *metA*, *ftsZ*, *relA* and *metG* alleles encoded by O26 : H11 STEC cumulatively distinguished this serotype from O157 : H7 (Gilmour *et al.*, 2005). The conservation of these loci between O26 : H11 strains, and the genetic distance from the other *E. coli* serotypes suggested that sequence-based typing of additional STEC might reveal serotype-specific alleles. In this study, additional DNA sequence data at these loci was obtained for a range of STEC and a single locus was observed to encode allelic variants correlating to individual STEC O-serogroups. We therefore present a simple molecular method for the identification of STEC serogroups, including both O157 and non-O157 strains.

## METHODS

### Bacterial strains.

STEC strains (Table 1) were obtained from the reference stocks of the Enteric Diseases Program at the National Microbiology Laboratory that originated from human sources at various Canadian provincial health laboratories during 1985–2005, or were recent clinical isolates obtained from the Alberta Provincial Laboratory for Public Health (nomenclature XX-YYYY, where XX generally refers to the year of isolation). During the course of these studies, five outbreak-associated STEC isolates were provided by Nova Scotia Public Health, Halifax, Nova Scotia, Canada. Confirmation of O : H serotype was completed with antisera prepared at the National Microbiology Laboratory (Ewing, 1986).

### PCR and sequencing.

Template DNA was prepared by centrifuging 1 ml exponential phase culture grown in brain heart infusion broth, resuspending the pellet in 1 ml TE buffer (Sigma; 10 mM Tris/HCl, 1 mM EDTA, pH 8.0) and boiling the cells for 15 min. Boiled cells were pelleted, and the supernatant was removed and used as the DNA template in PCR.

Oligonucleotide primers used to amplify segments of *mdh*, *gnd*, *gcl*, *ppk*, *metA*, *ftsZ*, *relA* and *metG* are presented in Table 2. PCR was performed with high fidelity Platinum *Taq* (Invitrogen), following the manufacturer’s directions. The thermocycling parameters for *fts*Z, *rel*A and *met*G included an initial denaturation at 94 °C for 5 min, 35 cycles of denaturation at 94 °C for 40 s, annealing at 50 °C for 45 s and extension at 68 °C for 45 s, with a final extension at 68 °C for 5 min. The annealing temperature for *met*A, *mdh*, *gcl* and *ppk* was 58 °C, and 52 °C for *gnd*. PCR products were purified using the QIAquick PCR purification kit (Qiagen) and sequenced using the same primers that generated these amplicons. Sequencing was performed on an ABI3730 (Applied Biosystems) and the data were deposited in GenBank with accession nos DQ472524–DQ472651. Existing genomic sequence data for *E. coli* O157 : H7 EDL933, O157 : H7 Sakai, O6 : H1 CFT073 and K-12 (GenBank accession nos NC_000913, BA000007, NC_002655, NC_004431) was included in our dataset for each of the above loci. From directed studies against the *gnd* locus (Tarr *et al.*, 2000; Paton & Paton, 1999b; Wang *et al.*, 1998), we included sequence data from O157 : H7 and O157 : NM (GenBank accession nos AF176359, AF176358, AF176357, AF176356, AF176360, AF176361 and AB008676), O113 : H2 (AF172324), O111 (AF078736) and non-toxin encoding O157 and O55 (AF176368, AF176367, AF176366, AF176363, AF176362, AF176369 and AF176373). Our previously acquired sequence data from O26 : H11, O26 : H6 and O26 : H32 strains were also included (GenBank accession nos AY973395–AY973421; Gilmour *et al.*, 2005).

### Bioinformatics.

Multiple sequence alignments were completed using ClustalW (www.ebi.ac.uk/clustalw/), neighbour-joining trees were constructed with Hasegawa–Kishino–Yano (HKY85) distance correction using SplitsTree4 (Huson, 1998), and genetic diversity statistics were calculated using DnaSP 4.10.3 (Rozas *et al.*, 2003). Pairwise global alignments were calculated using Align (www.ebi.ac.uk/emboss/align/#).

## RESULTS AND DISCUSSION

### Sequence typing correlates to O-antigen serogroups

**Fig. 1. f1:**
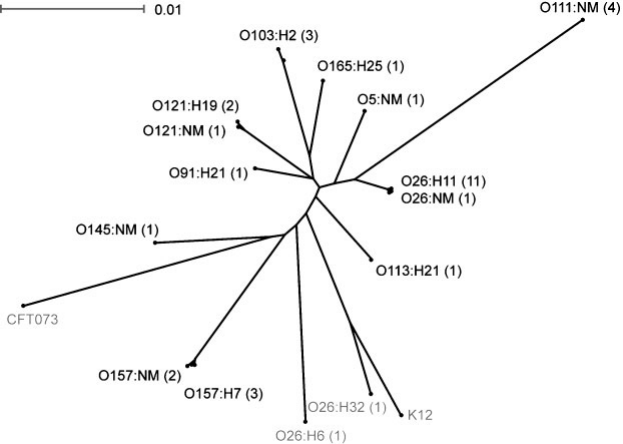
Phylogeny of the concatenated segments of *mdh*, *gnd*, *gcl*, *ppk*, *metA*, *ftsZ*, *relA* and *metG* encoded by *E. coli*. This is based upon a neighbour-joining tree constructed with Hasegawa–Kishino–Yano (HKY85) distance correction. Sequences obtained from GenBank are identified in Methods. The serotype of strain K-12 was not designated, and the serotype of uropathogenic strain CFT073 was O6 : K2 : H1. Shiga toxin-producing serotypes are indicated in black type, and strains not encoding *stx* are indicated in grey. The number of sequences per serotype is indicated in parentheses. Bar, scale of the distance score.

The alleles of *mdh*, *gnd*, *gcl*, *ppk*, *metA*, *ftsZ*, *relA* and *metG* encoded by O26 : H11 STEC cumulatively distinguished this serotype from O157 : H7 (Gilmour *et al.*, 2005), and the corresponding segments of these loci were sequenced for STEC serotypes O111 : NM, O113 : H21, O157 : NM, O145 : NM, O91 : H21, O121 : H19, O121 : NM, O103 : H2, O165 : H25 and O5 : NM. This panel of STEC strains included isolates from each of the most predominant O-serogroups and O : H-serotypes observed in Canada (Gilmour *et al.*, 2005, 2006), and amongst individual serotypes, strains with different *stx* genotypes were included when available (Table 1). This sequence dataset was compared to previously published sequence data for STEC serotypes O157 : H7 and O26 : H11, as well as non-toxin producing O26 : H32, O26 : H6, K12 and O6 : H1 (strain CFT073) strains using the 4464 nucleotide concatenate of the eight genetic determinants (Fig. 1). Each of the examined serogroups had distinct sequence types, including NM STEC strains of O121 and O157, were 99.8 and 99.9 % identical to O121 : H19 and O157 : H7 strains, respectively. The observed phylogenetic separation between serogroups, and homogeneity within strains of the same serogroup, indicated that these genetic traits have been acquired by and vertically inherited within individual STEC serogroup lineages.

### Molecular-based serogrouping with four loci

**Fig. 2. f2:**
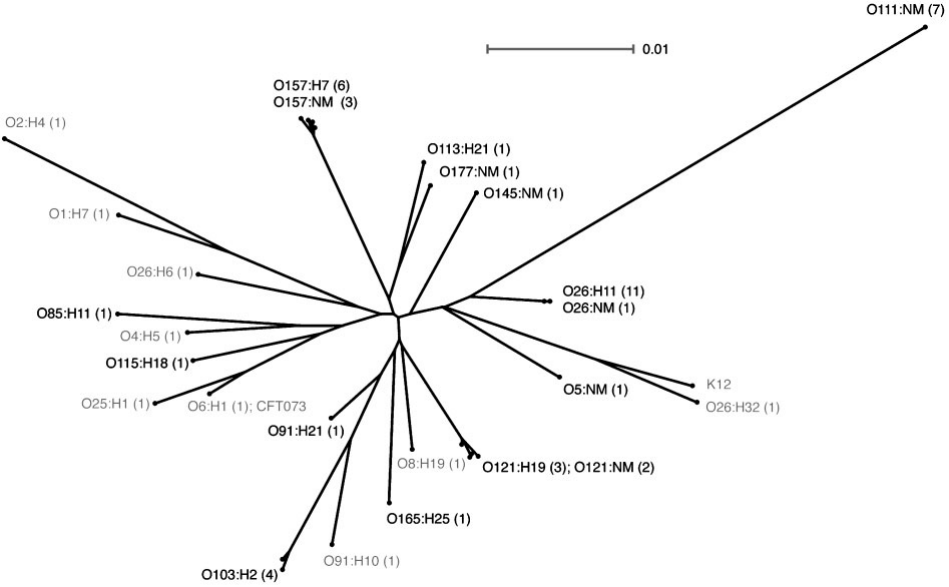
Phylogeny of the concatenated segments of *gnd*, *gcl*, *ppk* and *relA* encoded by *E. coli*. This is based upon a neighbour-joining tree constructed with Hasegawa–Kishino–Yano (HKY85) distance correction. Sequences obtained from GenBank are identified in Methods. Shiga toxin-producing serotypes are indicated in black type, and strains not encoding *stx* are indicated in grey. The number of sequences per serotype is indicated in parentheses. Bar, scale of the distance score.

Additional sequencing was performed at selected loci in an expanded panel of strains to determine if the phylogenetic separation observed between serogroups was maintained in a larger dataset (Table 1). The genetic determinants that contributed the majority of the observed genetic diversity (*gnd* and *gcl*; Table 3) or encoded putative serogroup-specific regions (*ppk* and *relA*; data not shown) were selected for further study. This panel included further strains from the serotypes represented in Fig. 1, as well as seropathotype D and non-toxin encoding *E. coli* strains recovered from paediatric stool samples (L. Chui, unpublished data). The overall genetic distinction between STEC serogroups (as determined in the eight locus scheme) was also represented amongst these four loci, and the additional strains and serogroups (Fig. 2).

### Molecular-based serogrouping with the *gnd* locus

**Fig. 3. f3:**
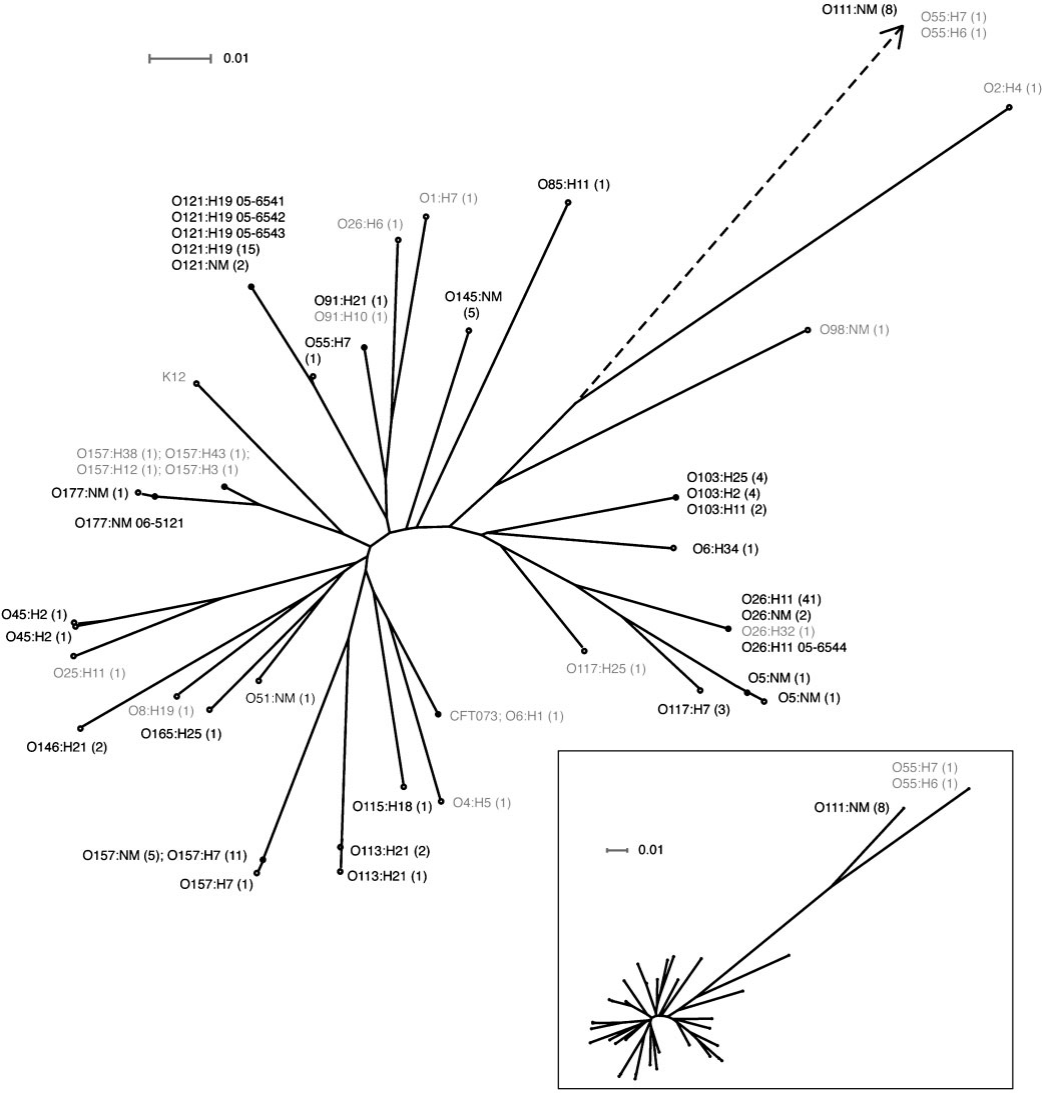
Phylogeny of the *gnd* locus encoded by *E. coli*. This is based upon a neighbour-joining tree constructed with Hasegawa–Kishino–Yano (HKY85) distance correction. Sequences obtained from GenBank are identified in Methods. Shiga toxin-producing serotypes are indicated in black type, and strains not encoding *stx* are indicated in grey. The number of sequences per serotype is indicated in parentheses. Strain identification numbers are indicated for outbreak-associated clinical isolates. The dotted line indicates outlying *gnd* sequences, which are presented in relation to the entire dataset in the inset. Bar, scale of the distance score.

The *gnd* locus was the most genetically diverse of all examined loci (Table 3), and notably, this determinant is immediately adjacent to the O-antigen gene cluster. Additional sequencing of the 643 bp region of *gnd* was performed (Table 1), and *gnd* sequence data available in GenBank for O157, O113 and O111 STEC, as well as non-toxin encoding O157 and O55 strains, were also included in comparative analyses. In total, *gnd* DNA sequences were collected from 144 strains and 26 O-serogroups (comprising 42 O : H-serotypes). The overall genetic distinction between serogroups (as determined in the eight and four loci schemes) was also represented in this single locus, as each examined STEC O-serogroup encoded a unique *gnd* allele (Fig. 3). For some of the most clinically significant STEC serogroups (O157, O26, O121, O145, O111 and O103) the *gnd* DNA sequences were compared between multiple strains (from 5 to 43 sequences), and for each serogroup all STEC strains encoded an identical *gnd* allele (Fig. 3). The only exception was O157 : H7 strain 87-16 (GenBank accession no. AF176360), which encoded a single nucleotide polymorphism compared to the other O157 strains, but otherwise the *gnd* alleles were conserved within STEC serogroup classifications. Furthermore, non-toxin encoding strains of O157, O26, O55, O6 and O117 encoded distinct *gnd* alleles compared to STEC strains of the same serogroup. Sequence typing of *gnd* was, therefore, a promising molecular method correlating minimally with the O-serogroup of clinical STEC strains. The O111 : NM STEC and non-toxin-producing O55 strains encoded *gnd* sequences outlying from the main cluster (Fig. 3) and these were homologous to *Citrobacter* spp. *gnd* alleles (Nelson & Selander, 1994). However, since pure bacterial isolates are preferred for preparation of DNA sequencing template, all isolates undergoing *gnd* DNA sequence-based serogrouping should previously be classified as STEC.

During the course of this study, outbreak-related isolates of non-O157 STEC were sent to the National Microbiology Laboratory for serotyping and genetic characterization. The *gnd* sequence data for each of isolates 05-6541 to 05-6543 clustered with known O121 strains (Fig. 3). A concurrent non-O157 sporadic isolate (05-6544) was also examined at *gnd* and this sequence clustered with known O26 : H11 strains (Fig. 3). Strain 06-5121 was isolated from a hospitalized patient with haemolytic uraemic syndrome and the *gnd* sequence of this strain was 99.8 % identical to a known O177 : NM isolate (Fig. 3). In correlation with these molecular data, subsequent serotyping using traditional methodologies characterized these isolates as O121 : H19, O26 : H11 and O177 : NM. The *gnd* DNA sequence-based serogrouping method therefore provided an advantageous alternative to O-specific immunoreagents during these crises. Over 55 serogroups of STEC have been reported to be associated with human disease (Johnson *et al.*, 2006), and an international panel of STEC strains from each serogroup, including the emerging sorbitol-fermenting O157, will be required to further validate this method.

The proportion of synonymous and nonsynonymous mutations was calculated for each locus from the accumulated DNA sequence data (Table 3). As expected for core loci, the majority of mutations were synonymous (dN/dS <1), but the *gnd* locus had the greatest number of nonsynonymous sites. This locus has already been identified as a polymorphic *E. coli* locus compared to other core loci (Bisercic *et al.*, 1991; Nelson & Selander, 1994; Dykhuizen & Green, 1991). A comparable ratio of synonymous versus nonsynonymous mutations was also reported by Bisercic *et al.* (1991). Genetic diversity at *gnd* arose in parallel to the extensive diversity and recombination that occurred at the neighbouring O-antigen gene cluster, and it is likely that these two genetic traits were co-inherited between lineages (Tarr *et al.*, 2000; Nelson & Selander, 1994). To our knowledge, there is no indication that O-serogroups that encode similar *gnd* alleles (e.g. STEC O121 and O55) also encode similar O-antigen gene clusters, nor are the antigens themselves similar. The potential utility of a locus subject to recombination between genera might be seemingly limited for the purpose of molecular-based serogrouping; however, we currently observed conserved STEC serogroup-specific genetic polymorphisms at the *gnd* locus. Between strains of an individual STEC O-serogroup we observed conserved *gnd* alleles, and no serogroup encoded a *gnd* allele that was identical to another serogroup. This study provides a simple method for molecular-based serogrouping of *E. coli* strains encoding *stx*, which can be detected by a wealth of molecular reagents (Gilmour *et al.*, 2006; Hsu *et al.*, 2005; Nielsen & Andersen, 2003; Reischl *et al.*, 2002; Wang *et al.*, 2002). This method was used to characterize O121 : H19, O26 : H11 and O177 : NM clinical isolates prior to serological confirmation during an outbreak investigation, and could, therefore, improve the scope of STEC molecular diagnostics beyond the O157 serogroup.

## Figures and Tables

**Table 1. t1:** Bacterial strains used in this study Strains characterized during outbreak investigations are identified (O).

Seropathotype*	**Serotype**	**Strain ID**	**Source†**	**Sequencing scheme‡**	***stx1***	***stx2***	**LEE§**	**Reference**
A	O157 : H7	87-1215	NML	8 loci	+	+	+	Gilmour *et al.* (2006)
	O157 : H7	01-8110	NML	4 loci	+	+	+	Gilmour *et al.* (2006)
	O157 : H7	05-0958	SK HPL	8 loci	−	+	+	Gilmour *et al.* (2006)
	O157 : H7	04-4319	SK HPL	4 loci	+	−	+	Gilmour *et al.* (2006)
	O157 : H7	03-2641	AB PLPH	4 loci	+	+	+	Gilmour *et al.* (2006)
	O157 : NM	01-6434	AB PLPH	8 loci	+	−	+	Gilmour *et al.* (2006)
	O157 : NM	03-3088	AB PLPH	4 loci	+	+	+	This study
	O157 : NM	03-5296	AB PLPH	8 loci	+	+	+	Gilmour *et al.* (2006)
B	O26 : H11	01-6372	NS PHL	8 loci	+	−	+	Gilmour *et al.* (2005)
	O26 : H11	03-2816	AB PLPH	8 loci	+	−	+	Gilmour *et al.* (2005)
	O26 : H11	05-6544	NS PHL (O)	*gnd*	+	−	+	This study
	O103 : H2	99-2076	BCCDC	8 loci	+	−	+	Gilmour *et al.* (2006)
	O103 : H2	04-2446	MB CPL	8 loci	+	−	+	Gilmour *et al.* (2006)
	O103 : H2	01-6102	SK HPL	8 loci	+	−	+	Gilmour *et al.* (2006)
	O103 : H2	03-3967	AB PLPH	4 loci	+	−	+	This study
	O103 : H11	04-3973	MB CPL	*gnd*	+	−	+	Thompson *et al.* (2005)
	O103 : H11	06-4464	MB CPL	*gnd*	+	−	+	This study
	O103 : H25	03-1028	MB CPL	*gnd*	+	−	+	Thompson *et al.* (2005)
	O103 : H25	03-1030	MB CPL	*gnd*	+	−	+	Thompson *et al.* (2005)
	O103 : H25	04-3972	MB CPL	*gnd*	+	−	+	Thompson *et al.* (2005)
	O103 : H25	03-2444	MB CPL	*gnd*	+	−	+	Thompson *et al.* (2005)
	O111 : NM	03-3991	AB PLPH	4 loci	+	−	+	Gilmour *et al.* (2006)
	O111 : NM	04-3794	MB CPL	8 loci	+	+	+	Gilmour *et al.* (2006)
	O111 : NM	98-8338	BCCDC	4 loci	+	−	+	Gilmour *et al.* (2006)
	O111 : NM	00-4748	SK HPL	8 loci	+	+	+	Gilmour *et al.* (2006)
	O111 : NM	00-4440	BCCDC	4 loci	+	−	+	Gilmour *et al.* (2006)
	O111 : NM	01-0252	BCCDC	8 loci	+	+	+	Gilmour *et al.* (2006)
	O111 : NM	01-1215	BCCDC	8 loci	+	−	+	Gilmour *et al.* (2006)
	O121 : H19	03-2636	AB PLPH	4 loci	−	+	+	Gilmour *et al.* (2006)
	O121 : H19	03-2642	AB PLPH	*gnd*	−	+	+	Gilmour *et al.* (2006)
	O121 : H19	03-2832	AB PLPH	8 loci	−	+	+	Gilmour *et al.* (2006)
	O121 : H19	05-6541	NS PHL (O)	*gnd*	−	+	+	This study
	O121 : H19	05-6542	NS PHL (O)	*gnd*	−	+	+	This study
	O121 : H19	05-6543	NS PHL (O)	*gnd*	−	+	+	This study
	O121 : H19	00-5288	BCCDC	8 loci	−	+	+	Gilmour *et al.* (2006)
	O145 : NM	03-4699	AB PLPH	8 loci	+	−	+	Gilmour *et al.* (2006)
	O145 : NM	04-7099	MB CPL	*gnd*	+	−	+	This study
	O145 : NM	04-7194	MB CPL	*gnd*	+	−	+	This study
	O145 : NM	04-1449	MB CPL	*gnd*	+	−	+	This study
	O145 : NM	03-6430	MB CPL	*gnd*	+	−	+	Thompson *et al.* (2005)
	O145 : NM	02-5149	BCCDC	*gnd*	+	−	+	This study
C	O5 : NM	03-2825	AB PLPH	8 loci	+	−	+	Gilmour *et al.* (2006)
	O5 : NM	03-2682	MB CPL	*gnd*	+	−	+	Thompson *et al.* (2005)
	O91 : H21	85-489	NML	8 loci	−	+	−	Gilmour *et al.* (2006)
	O113 : H21	93-0016	NML	8 loci	−	+	−	Gilmour *et al.* (2006)
	O113 : H21	04-1450	MB CPL	*gnd*	−	+	−	Thompson *et al.* (2005)
	O121 : NM	99-4389	NML	8 loci	−	+	+	Gilmour *et al.* (2006)
	O121 : NM	03-4064	AB PLPH	4 loci	−	+	+	This study
	O165 : H25	00-4540	BCCDC	8 loci	−	+	+	Gilmour *et al.* (2006)
D	O6 : H34	03-5166	MB CPL	*gnd*	−	+	−	Thompson *et al.* (2005)
	O45 : H2	05-6545	NS PHL	*gnd*	+	−	+	This study
	O45 : H2	04-2445	MB CPL	*gnd*	+	−	+	Thompson *et al.* (2005)
	O55 : H7	05-0376	NML	*gnd*	+	−	+	This study
	O85 : H1	03-3638	AB PLPH	4 loci	−	+	−	This study
	O115 : H18	03-3645	AB PLPH	4 loci	+	+	−	This study
	O117 : H7	05-0379	NML	*gnd*	+	−	−	This study
	O117 : H7	02-0035	BCCDC	*gnd*	+	−	−	This study
	O117 : H7	02-4495	BCCDC	*gnd*	+	+	−	This study
	O146 : H21	02-7808	BCCDC	*gnd*	+	−	−	This study
	O146 : H21	02-1628	BCCDC	*gnd*	+	−	−	This study
	O177 : NM	03-3974	AB PLPH	4 loci	−	+	+	This study
	O177 : NM	06-5121	NS PHL (O)	*gnd*	−	+	+	This study
na	O1 : H7	03-3964	AB PLPH	4 loci	−	−	−	This study
	O2 : H4	03-2815	AB PLPH	4 loci	−	−	−	This study
	O4 : H5	03-3266	AB PLPH	4 loci	−	−	−	This study
	O6 : H1	03-2638	AB PLPH	4 loci	−	−	−	This study
	O8 : H19	03-2639	AB PLPH	4 loci	−	−	−	This study
	O25 : H1	03-2637	AB PLPH	4 loci	−	−	−	This study
	O26 : H6	01-5872	MB CPL	8 loci	−	−	−	Gilmour *et al.* (2005)
	O26 : H32	99-4328	SK HPL	8 loci	−	−	−	Gilmour *et al.* (2005)
	O51 : NM	04-2640	MB CPL	*gnd*	−	−	−	This study
	O91 : H10	03-3269	AB PLPH	4 loci	−	−	−	This study
	O98 : NM	02-7464	NB PHL	*gnd*	−	−	−	This study
	O117 : H25	02-0714	NB PHL	*gnd*	−	−	−	This study

*na, Not applicable. Strains that do encode *stx* are not classified in the seropathotype scheme (Karmali *et al.*, 2003). †AB PLPH, Alberta Provincial Laboratory for Public Health; BCCDC, British Columbia Centre for Disease Control; MB CPL, Manitoba Cadham Provincial Laboratory; NML, National Microbiology Laboratory standard strain; NB PHL, New Brunswick Public Health Laboratory; NS PHL, Nova Scotia Public Health Laboratory; SK HPL, Saskatchewan Health Provincial Laboratory. ‡DNA sequencing was performed for 8 loci (*mdh*, *gnd*, *gcl*, *ppk*, *metA*, *ftsZ*, *relA* and *metG*), 4 loci (*gnd*, *gcl*, *ppk* and *relA*) or solely the *gnd* locus. §As determined by PCR screening for the *espZ* gene (Gilmour *et al.*, 2006).

**Table 2. t2:** Oligonucleotides used in this study

**Oligonucleotide**	**Target**	**Sequence (5′ to 3′)**	**Product size (bp)**	**Reference**
GIL213	*ftsZ*	GATCACTGAACTGTCCAAGCATG	450	Gilmour *et al.* (2005)
GIL214	*ftsZ*	TCAAGAGAAGTACCGATAACCAC		
gcl-F	*gcl*	GCGTTCTGGTCGTCCGGGTCC	758	Adiri *et al.* (2003)
gcl-R	*gcl*	GCCGCAGCGATTTGTGACAGACC		
gnd-F	*gnd*	GGCTTTAACTTCATCGGTAC	712	Noller *et al.* (2003)
gnd-R	*gnd*	TCGCCGTAGTTCAGATCCCA		
mdh-F	*mdh*	CAACTGCCTTCAGGTTCAGAA	580	Noller *et al.* (2003)
mdh-R	*mdh*	GCGTTCTGGATGCGTTTGGT		
metA-F	*metA*	CGCAACACGCCCGCAGAGC	601	Adiri *et al.* (2003)
metA-R	*metA*	GCCAGCTCGCTCGCGGTGTATT		
GIL219	*metG*	TGGCTGACCCGCAGTTGTAC	503	Gilmour *et al.* (2005)
GIL220	*metG*	GGTCAACTTTGGCGAAGTCGTC		
ppk-F	*ppk*	TGCCGCGCTTTGTGAATTTACCG	758	Adiri *et al.* (2003)
ppk-R	*ppk*	CCCCGGCGCAGAGAAGATAACGT		
GIL215	*relA*	TCTGTTTCCTCCGAACAGGTCG	470	Gilmour *et al.* (2005)
GIL216	*relA*	ACAATACGTACCGCACGCACATC		

**Table 3. t3:** Genetic diversity of the protein-encoding loci of *E. coli* sequenced in this study For comparative purposes, multiple statistics for the *gnd* locus are presented as increasing numbers of serotypes and strains were analysed.

**Target**	No. of sequences*	**No. of serotypes†**	**Size of target (bp)**	**No. of polymorphic sites (*π*)‡**	**No. of synonymous polymorphic sites**	**No. of nonsynonymous polymorphic sites**	**dN/dS§**
*gnd*	47	42	643	210 (0.067)	189	21	0.035
	27	26	643	198 (0.061)	179	19	0.028
	17	16	643	173 (0.062)	154	19	0.030
*gcl*	26	26	654	68 (0.023)	61	7	0.023
*relA*	30	26	425	42 (0.019)	41	1	0.002
*mdh*	18	16	644	31 (0.010)	28	3	0.018
*ftsZ*	16	16	404	17 (0.010)	17	0	0.000
*metA*	16	16	559	36 (0.015)	29	7	0.115
*metG*	16	16	434	46 (0.024)	42	4	0.021
*ppk*	28	26	701	40 (0.013)	39	1	0.005

*Identical DNA sequences belonging to the same O : H serotype were included only once. †Minimally includes the serotypes indicated in Fig. 1 (when no. of serotypes=16), in Fig. 2. (when no. of serotypes=26) or in Fig. 3 (when no. of serotypes=42). ‡*π*, Measure of genetic diversity. §Rate of nonsynonymous and synonymous mutations.

## Abbreviations

- LEE, locus for enterocyte effacement
- NM, non-motile
- STEC, Shiga toxin-producing *Escherichia coli*
